# Editorial: Recent advances in bioceramics for health

**DOI:** 10.3389/fbioe.2023.1264799

**Published:** 2023-08-01

**Authors:** Simone Sprio, Iulian Antoniac, Jerome Chevalier, Michele Iafisco, Monica Sandri, Anna Tampieri

**Affiliations:** ^1^ Institute of Science, Technology and Sustainability for Ceramics, National Research Council of Italy (ISSMC-CNR, former ISTEC-CNR), Faenza, Italy; ^2^ Faculty of Material Science and Engineering, University Politehnica of Bucharest, Bucharest, Romania; ^3^ MATEIS, CNRS, Université de Lyon, INSA de Lyon, Université Claude Bernard Lyon 1, Villeurbanne, France

**Keywords:** bioceramics, calcium phosphates, bio-glasses, bone regeneration, skin regeneration

For at least half a century intensive effort has been dedicated to the design and development of inorganic materials functioning as implants to repair damages due to traumas or degenerative pathologies. Particularly, ceramic materials and bio-glasses have been intensively explored for their applicative potential not only with the aim to repair tissues and organs but, ultimately, to regenerate missing tissue regions. In this respect, pushed by ever raising clinical needs with great socio-economic impact, along the recent decades the main focus of scientific research moved from the study of bio-inert materials, designed to function as tissue substitutes but without any relevant bio-functionality, to the development of bioactive materials and devices. Such constructs are designed to exhibit low chemical stability and suitable nanostructure, thus being able to exchange information with cells and activate the natural physiological processes that can lead to the formation of new healthy and mechanically functional tissue. Moreover, recently the focus of research is increasingly directed towards the development of solutions capable of contrasting the insurgence of infections, which is among the most serious concerns in medicine and a major hindrance to a positive clinical outcome, also due to the ever-increasing bacterial resistance to antibiotics.

**FIGURE 1:**
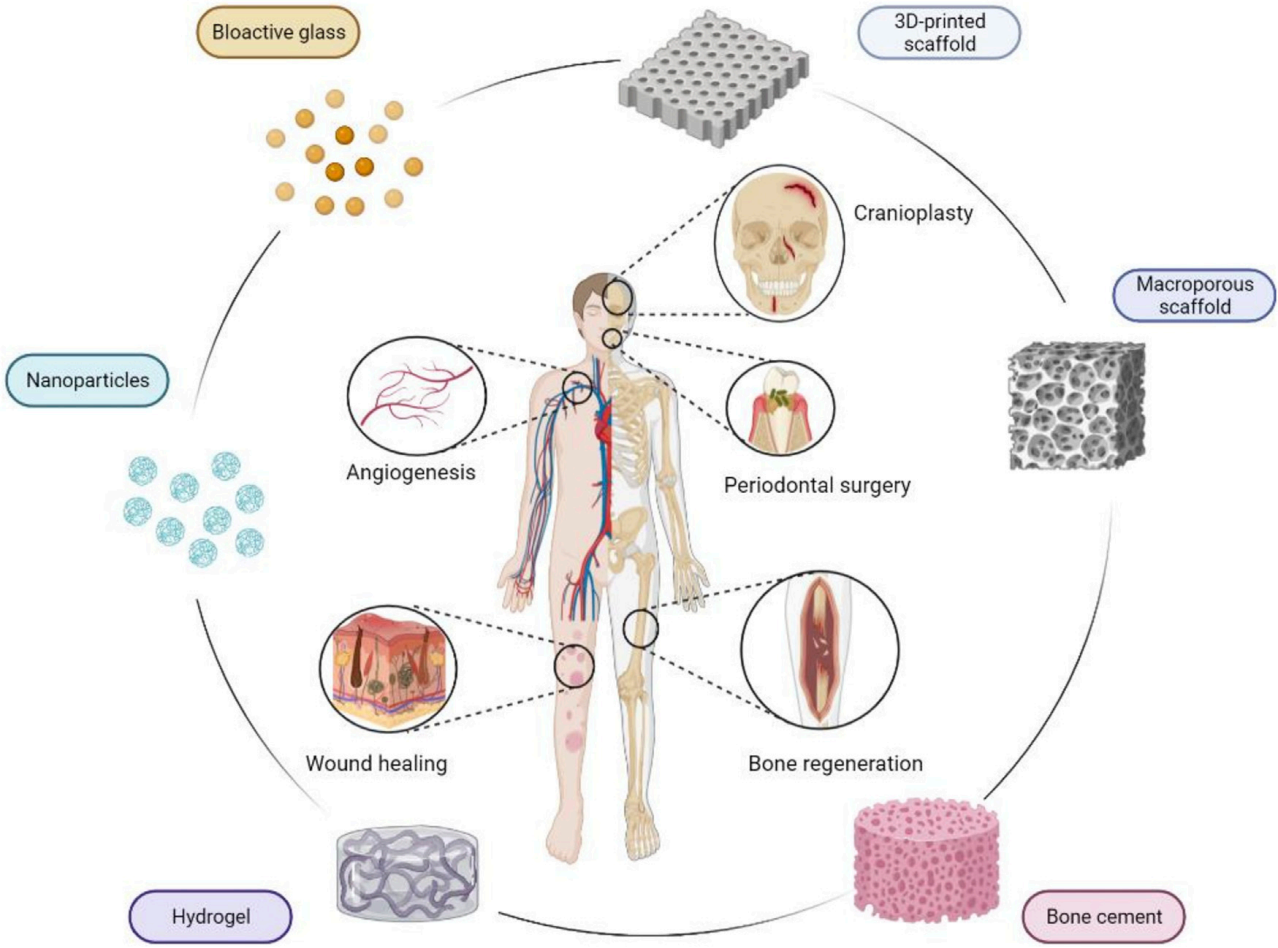
Pictorial scheme of bioceramics and bioglasses and their application in regenerative medicine.

As far as ceramic and hybrid materials (these obtained by nucleation of inorganic nanophases on templating organic biomolecules) are concerned, their main application has always targeted the regeneration of hard connective tissues such as bone and teeth, due to their compositional affinity with compounds such as calcium phosphates and bio-glasses. In this regard, the development of novel synthesis methods, processes and additive manufacturing technologies are directed towards the creation of bioactive, nanostructured 3D constructs able to self-consolidate and/or with customized architecture and geometry, targeting application to different anatomical districts such as long bones, spine, maxilla and the skull. In recent years however, bioactive ceramics and hybrids are increasingly being investigated for new applications to soft tissues such as the skin, thanks to their bio-resorption and ion exchange ability which allows for the creation of a local microenvironment favorable to regenerative processes and aiding to prevent the insurgence of infections (Figure 1).

In this scenario, the present Article Research Topic includes contribution from participants to the Bioceramics 32 conference, the Annual Meeting of the International Society of Ceramics in Medicine, bringing together interdisciplinary contributions on recent developments in biomaterial science and tissue engineering approaches within all these fields of application, in some cases corroborated by *in vivo* pre-clinical and clinical tests.

In particular, Knabe et al. describes the development of a 3D printed calcium silicon orthophosphate construct enriched with stem cells and with arteriovenous bundles to promote osteogenesis and angiogenesis in the regeneration of segmental maxillofacial defects. The work highlights the relevance of achieving extensive angiogenesis to ensure bone regeneration in the whole bone defect. Vascularization is a major problem in large bone reconstruction and the present study validated the tissue engineering approach in small animal model.

The use of ionic substitutes in bioceramics to increase bioactivity and to promote metabolically-driven scaffold resorption is a topic of major interest. In this regard, Dapporto et al. described the development of nanostructured apatite bone cements, partially replaced with strontium ions to modulate the physiological bone turnover, and functionalized to permit the release of drugs with prolonged and sustained kinetics over time, potentially useful against infections and tumors. The work also highlighted the intrinsic antibacterial properties of nanostructured materials which may be relevant in contrasting microbial resistance to antibiotics. On the other hand, Yoo et al. describe a bone substitute based on biphasic calcium phosphate (i.e., hydroxyapatite + tricalcium phosphate) partially substituted with lithium ions. Previous studies reported the positive effect of lithium as well as other doping ions on the osteogenesis process, however there is still little knowledge on dose-related effects, particularly for ions such as lithium, not strictly related to the bone physiology. The present work identified threshold limits for lithium substitution above which phase destabilization occurred, leading to burst release of Li ions in turn reducing the osteogenic differentiation of human dental pulp stem cells.

Bioactive ceramics, as well as hybrid apatite-collagen constructs, are highly studied for vertebral regeneration. In this regard Griffoni et al. describes the use of apatite nanoparticles partially substituted with magnesium ions, developed in the form of an extrudable paste for postero-lateral fusion in the treatment of degenerative spine diseases. This work was validated by clinical studies, and gives a promising perspective for the use of synthetic bioactive bone substitutes in spinal fusion procedures, in the place of iliac crest bone graft which is today the golden standard but is affected by various drawbacks and complications.

In another paper Ryu et al. describe a clinical study highlighting the use of bioglasses for the fabrication of spacers for anterior cervical discectomy and fusion surgery which were here compared with allografts. The results are very promising, showing excellent osteointegration and absence of any failures after 60 months. Hence, also this clinical study highlights the relevance of synthetic bioactive substitutes in the place of allografts, to promote bone tissue regeneration.

Bioglasses, inorganic amorphous substances capable of dissolving in the physiological environment whereas releasing biologically active ions, are gaining increasing interest for applications in skin care. In this regard, Solanki et al. describes the use of bioglass fibers capable of releasing cobalt ions in quantities useful for the activation of specific molecular pathways relevant for angiogenesis. The work specifically demonstrated the positive effect of cobalt and other bioactive ions, when slowly released from the bioglass, in the promotion of vascular endothelial growth factors thus highlighting the potential of bioglasses to treat chronic wounds. In a similar work, Elshazly et al. employed boron-enriched bioglass nanofibers for the regeneration of full-thickness skin defects on rabbits. This work remarked the dissolution ability of bioglasses, as well as the relevance of released ions to enhance the angiogenic, regenerative and antibacterial ability.

The works published in the present Research Topic are referred to different anatomical districts, each of them requiring specific design of biomaterials chemistry and structure, therefore the Research Topic can be a useful reference for the reader, particularly addressing early stage researchers in the field of biomaterials development.

